# Objective estimation of m-CTSIB balance test scores using wearable sensors and machine learning

**DOI:** 10.3389/fdgth.2024.1366176

**Published:** 2024-04-19

**Authors:** Marjan Nassajpour, Mustafa Shuqair, Amie Rosenfeld, Magdalena I. Tolea, James E. Galvin, Behnaz Ghoraani

**Affiliations:** ^^1^^ Department of Computer and Electrical Engineering and Computer Science, Florida Atlantic University, Boca Raton, FL, United States; ^^2^^ Department of Neurology, Comprehensive Center for Brain Health, University of Miami, Boca Raton, FL, United States

**Keywords:** balance assessment, wearable sensors, machine learning, m-CTSIB, XGBOOST

## Abstract

Accurate balance assessment is important in healthcare for identifying and managing conditions affecting stability and coordination. It plays a key role in preventing falls, understanding movement disorders, and designing appropriate therapeutic interventions across various age groups and medical conditions. However, traditional balance assessment methods often suffer from subjectivity, lack of comprehensive balance assessments and remote assessment capabilities, and reliance on specialized equipment and expert analysis. In response to these challenges, our study introduces an innovative approach for estimating scores on the Modified Clinical Test of Sensory Interaction on Balance (m-CTSIB). Utilizing wearable sensors and advanced machine learning algorithms, we offer an objective, accessible, and efficient method for balance assessment. We collected comprehensive movement data from 34 participants under four different sensory conditions using an array of inertial measurement unit (IMU) sensors coupled with a specialized system to evaluate ground truth m-CTSIB balance scores for our analysis. This data was then preprocessed, and an extensive array of features was extracted for analysis. To estimate the m-CTSIB scores, we applied Multiple Linear Regression (MLR), Support Vector Regression (SVR), and XGBOOST algorithms. Our subject-wise Leave-One-Out and 5-Fold cross-validation analysis demonstrated high accuracy and a strong correlation with ground truth balance scores, validating the effectiveness and reliability of our approach. Key insights were gained regarding the significance of specific movements, feature selection, and sensor placement in balance estimation. Notably, the XGBOOST model, utilizing the lumbar sensor data, achieved outstanding results in both methods, with Leave-One-Out cross-validation showing a correlation of 0.96 and a Mean Absolute Error (MAE) of 0.23 and 5-fold cross-validation showing comparable results with a correlation of 0.92 and an MAE of 0.23, confirming the model’s consistent performance. This finding underlines the potential of our method to revolutionize balance assessment practices, particularly in settings where traditional methods are impractical or inaccessible.

## 1 Introduction

Balance is frequently used among healthcare professionals in various clinical settings, often associated with stability and postural regulation (1). Maintaining balance is critical in an individual’s functional status and safety. Balance may be impacted by various factors, including diseases, acute and chronic neurogenic injuries, and the natural aging process (2, 3). Assessing motor performance, which includes gait and stability, offers a valuable clinical approach for predicting a range of health implications. These include the risk of falls, risk of hospitalization, the onset of neurological disorders such as Parkinson’s disease, cognitive decline, and even mortality (4, 5). Recent research has shown that balance and gait disorders are prevalent among individuals with different forms of dementia, including Alzheimer’s disease (AD) and even in its early stages, such as mild cognitive impairment (MCI) (6). These disorders can considerably affect cognitive and functional abilities (7), leading to challenges in daily activities for adults with dementia, such as self-care, home maintenance, walking, and driving (8–10). These findings underscore the crucial need to assess and measure balance among adults.

There are various methods and tools used to measure balance. Several clinical assessments rely on visual examination by healthcare professionals, such as the Romberg test and Berg Balance Scale (BBS), or self-administered questionnaires like the Activities-specific Balance Confidence Scale (11–13). The Romberg test assesses balance by having the individual stand with feet together and arms at their side or crossed in front, first with eyes open and then closed. However, this test only evaluates balance on a stable surface and may not reflect the challenges encountered in dynamic environments. BBS, which includes 14 functional activities, provides a broader assessment but necessitates more extended periods and specialized expertise for administration. The Timed Up and Go (TUG) test is another test that measures mobility and requires a clear path of three meters, limiting its applicability in space-constrained environments. Moreover, it primarily focuses on the duration to complete the task rather than the quality of movement and balance during the performance (14). Self-administered questionnaires like the Activities-specific Balance Confidence Scale offer subjective self-assessment, which can be influenced by an individual’s perception and may not accurately represent actual balance abilities.

The Modified Clinical Test for Sensory Interaction and Balance (m-CTSIB) is a dynamic assessment tool that evaluates how individuals utilize their sensory systems to maintain balance. Distinct from the Romberg test, BBS, and TUG, the m-CTSIB adds complexity by including conditions that test balance with both eyes open and closed and on solid and compliant surfaces. Furthermore, the m-CTSIB can be completed quickly, typically less than a minute. Its rapid execution, coupled with its comprehensive nature, enhances its practical utility in various clinical settings. The m-CTSIB’s design to challenge multiple sensory inputs is not only more reflective of real-world scenarios where individuals must maintain balance with varying sensory feedback but also allows for early detection of balance impairments and the facilitation of targeted rehabilitation plans. For example, in older adults with Alzheimer’s disease, condition four of the m-CTSIB, which assesses balance with visual input removed and standing on a compliant surface, has significantly impacted functional capacity, highlighting its utility in this population (15, 16). Similarly, for individuals with idiopathic Parkinson’s disease, there has been evidence of the test’s validity, with accelerometer data from the m-CTSIB showing consistency with force plate measurements, reinforcing its application for these patients (17–19). Similar to the Romberg test, BBS, and TUG, clinicians evaluate m-CTSIB visually as the duration of the tests.

A significant limitation of these methods is their dependence on the clinician’s expertise, which can lead to variability in results. Moreover, these tests often only consider the duration of the test as the final measure, potentially overlooking crucial aspects of balance and stability. This limitation can result in inconsistent construct and criterion validity, varying based on the patient population and the method of administration, thus highlighting the need for more objective and comprehensive assessment tools in balance evaluation.

Employing recording instruments, such as Falltrak II (MedTrak VNG, Inc.), for assessing m-CTSIB introduces a systematic balance quantification, examining the integration of somatosensory, visual, and vestibular inputs. Falltrak II measures deviations of the center of pressure (COP) from the center of mass (COM), thereby offering a comprehensive and objective analysis of an individual’s postural stability (20). This objective quantification enhances the precision of m-CTSIB, rendering it a more exact tool for conducting detailed assessments of balance (21, 22). However, their primary limitation lies in the reliance on specialized, expensive equipment, which may not be readily accessible in all clinical settings. Additionally, these instruments often lack the flexibility for remote assessments, limiting their application in home or community-based healthcare scenarios where such evaluations are increasingly necessary.

In response, recent research has focused on integrating wearable sensor technology and machine learning algorithms to improve the accuracy and accessibility of balance assessments. Wearable sensors also offer a practical and cost-effective solution for capturing detailed movement data, essential for balance analysis. Positioned on areas like the lower back and lower limbs, these sensors provide insights into three-dimensional movement dynamics, essential for applications such as fall risk assessment in diverse populations. Coupled with the evolution of machine learning, these sensor-derived datasets transform into objective, quantifiable balance metrics, utilizing an array of machine learning techniques. For example, research by Bhargava et al. showcased the potential of using wearable coupled with machine learning to discern individuals with balance impairments from those without (23). Similarly, LeMoyne et al.’s work with support vector machines (SVM) and neural networks offered new insights into the gait characteristics of individuals with Friedreich’s ataxia compared to healthy controls (24). Howcroft et al. employed wearable sensors to classify fall risk in older adults, with SVM and neural networks emerging as the most effective intelligent modeling techniques for this purpose (25). Other examples include the objective assessment of TUG (26) and the approximation of BBS scores (27) using wearable sensors and machine learning. Please refer to (28, 29) for a detailed review of recent advancements in wearable sensor technology and machine learning for balance assessment.

Despite these advancements, a significant gap remains in the objective assessment of m-CTSIB scores without relying on specialized equipment like the Falltrak II. This dependency restricts access and complicates implementation in remote or underserved areas. Our study addresses this gap by introducing a novel approach using wearable sensors and machine learning to estimate m-CTSIB scores. By replacing the specialized force plate equipment, our approach significantly contributes to balance assessment by making it more accessible, cost-effective, and capable of remote administration. Such innovation would extend the benefits of comprehensive balance evaluation to a broader range of clinical and everyday settings.

Our methodology involved collecting motion data from 34 participants under four different sensory conditions of m-CTSIB using an array of inertial measurement unit (IMU) sensors complemented by a specialized system (Falltrak II) for precise m-CTSIB score evaluation. The wearable sensor data served as the input for our machine-learning models, and the corresponding m-CTSIB scores from Falltrak II acted as the ground truth labels for model training and validation. Multiple machine-learning models were then developed to estimate m-CTSIB scores from the wearable sensor data. Additionally, we explored the most effective sensor placements to optimize balance analysis. This novel approach represents a significant advance in objective balance assessment, especially valuable for remote monitoring in home-based or nursing care settings, potentially transforming balance disorder management. Our study addresses a crucial gap in balance assessment and sets a new benchmark in the application of wearable technology and machine learning in healthcare.

## 2 Materials and methods

**Figure 1:**
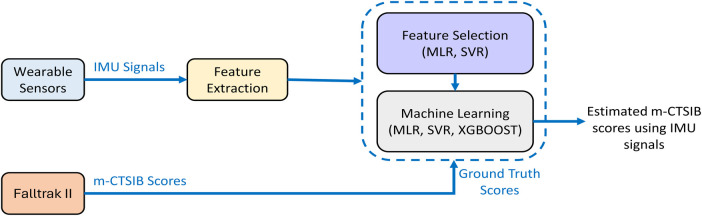
The diagram illustrates the proposed approach for estimating the m-CTSIB scores from wearable sensor data. m-CTSIB scores obtained in parallel through the Falltrak II system serve as the reference for training and testing our machine learning algorithms.

In this section, we detail our comprehensive approach encompassing data collection, signal processing techniques, and the application of machine learning methodologies. Figure 1 illustrates the key steps involved in our data processing and machine learning approach. Our system utilizes data from wearable sensors and Falltrak II scores as input and ground truth scores, respectively. The following sections provide a detailed description of each step in the process.

### 2.1 Subjects

The study recruited 34 participants, 12 males and 23 females, aged 21–88 years (see Table 1). The study adhered to the principles of the Helsinki Declaration and was approved by the Institutional Review Board (IRB). Informed consent was obtained from each participant through signed consent forms.

**Table 1:** Participant demographics.

Total participants	34	Right-handed	31
Gender (male, female)	12, 22	Hight (inches)	65.31±3.72
Age range (year)	58.78±18.06	Weight (pound)	169.34±45.88

Values are presented as numbers, mean ± SD, and/or [range].

#### 2.1.1 Participant selection criteria and population comparability analysis

The participant cohort for our study was selected to align with the demographic that commonly undergoes the m-CTSIB. Focused on community-dwelling adults and older adults, our inclusion criteria spanned those without cognitive deficits to those with MCI and early-stage AD, deliberately excluding individuals with more advanced AD. This choice was informed by evidence pointing to the m-CTSIB’s reliability in populations with mild cognitive variations and its capacity to offer significant insights into balance and mobility (30). The demographic inclusivity ensures our findings apply to the broader clinical and research contexts where the m-CTSIB is an established tool for balance assessment.

### 2.2 Recording tools

The Falltrak II system by MedTrak VNG, Inc., along with a series of IMU sensors, constituted the primary recording tools in this study. The Falltrak II system featured a pressure-sensitive platform that measured shifts in COP, both anterior-posterior (AP) and medial-lateral (ML). It provided a measure of the path length (PL) and average velocity (AV) of the COP. The units for PL are inches, representing the distance traveled by the COP, while AV is measured in inches per second, representing the average velocity of the COP movement. In addition to the Falltrak II system, IMU sensors, which included two Shimmer sensors and six APDM sensors, were utilized to capture comprehensive accelerometer and gyroscope data. The sensors’ technical specifications closely match, ensuring data consistency for our study. Table 2 compares the APDM and Shimmer sensors directly, both operating at 128 Hz with a range of ±16 g across three axes, facilitating robust comparative analysis. Section S2 in the Supplementary Material provides more details about APDM and Shimmer sensors. A microphone attached to the participants’ chest was also used for audio cues, essential for data segmentation across different experiments as elaborated in Section 2.6. Section S3 in the Supplementary Material provides more details on the measurement framework provided by the FallTrak II system.

**Table 2:** Specifications of APDM and shimmer sensors used in the study.

	APDM (31)	Shimmer (32)
Axes	3 axes	3 axes
Noise	$120\;\mu g/\sqrt{\mathrm{Hz}}$	$125\;\mu g/\sqrt{\mathrm{Hz}}$
Sample rate	128 Hz	128 Hz
Range	±16	±16
Resolution	17.5 bits	16 bits

### 2.3 Wearable sensor placements

**Figure 2:**
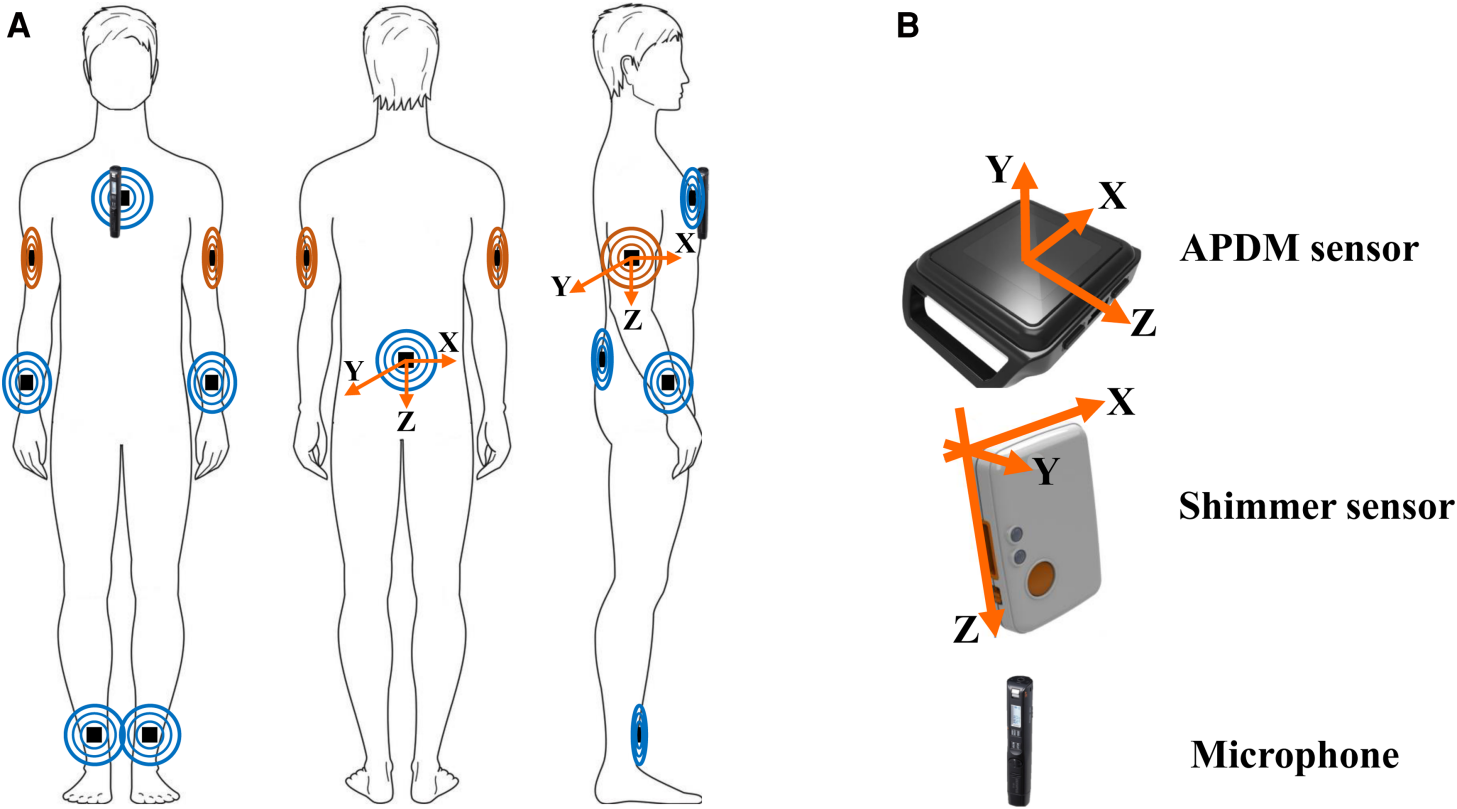
Sensor placement and orientation. (**A**) Shows the placement of APDM (in blue) and Shimmer (in orange) sensors, along with a microphone, on the human body. (**B**) Provides a detailed view of the sensors’ orientation, indicating the axes’ alignment for accurate data capture.

IMU sensors were placed on the participants to gather accelerometer and gyroscope data (see Figure 2A). The Shimmer sensors were placed on the upper arms, positioned just outside and below the deltoid muscle–the primary muscle shaping the contour of the shoulder. This specific placement was chosen to ensure central alignment and effective capture of upper body movements, facilitating detailed analysis of arm and shoulder dynamics crucial for understanding overall body sway. The APDM sensors were placed at several key points on the body for comprehensive motion analysis. One sensor was placed on each ankle, centered to track lower limb movements. This location is important for assessing leg stability and the role of the lower extremities in balance maintenance. Another sensor was secured on the lumbar region, specifically centered at the L3 vertebra. This placement is key for monitoring core body movements, offering valuable data on how the body’s midsection, an area pivotal for balance, responds to different postural demands. The sternum sensor was affixed to the flat surface of the chest, positioned just below the meeting point of the collar bones, ensuring it was centered for optimal data capture of the torso’s movements, contributing to our comprehension of how central body motion impacts balance. Lastly, sensors were placed on the wrists, akin to wearing a watch, to monitor wrist and hand movements, providing insights into the fine motor adjustments made by the participants to maintain balance. The careful positioning of these sensors ensured accurate and reliable data collection in our study.

Sensor placement was conducted by trained research personnel adhering to a standardized protocol, utilizing specific anatomical landmarks to ensure consistent participant positioning. We used detailed visual and written guidelines and preparatory practice sessions for the team to minimize variability in sensor attachment. Anatomical reference points, such as the deltoid muscle for the upper arms and the L3 vertebra for the lumbar region, were crucial for achieving uniformity. Furthermore, the team regularly reviewed and calibrated their techniques based on feedback, ensuring the accuracy and reliability of data collection were maintained throughout the study. This rigorous methodology was pivotal in addressing potential placement variability between subjects, thereby enhancing the study’s overall data integrity.

The consistent coordinate direction was established for all sensors to analyze and compare the recorded signals (see Figure 2B). The APDM sensors’ *Y*-axis was oriented away from the skin, and the *X*-axis followed the right-hand rule. The *Z*-axis, defined by the buttons on the sensor sides, faced the ground. For the Shimmer sensors, the port side of each sensor was oriented away from the ground, with the *Y*-axis facing outward from the skin. The *X*-axis was aligned according to the right-hand rule. All sensors, except those placed on the arms, recorded data from the accelerometer and gyroscope in three dimensions (*X*, *Y*, and *Z*), with the *X*-axis representing ML displacement, the *Y*-axis indicating AP displacement, and the *Z*-axis aligning with VT (vertical) motion. Due to the standing position, the orientation was adjusted for the sensors placed on the arms. The *X*-axis represented AP displacement, and the *Y*-axis indicated ML displacement.

### 2.4 Data synchronization

To ensure the accuracy and consistency of data across all sensors, all sensors were set to a uniform sampling frequency of 128 Hz and were synchronized. The APDM sensors were synchronized during their calibration phase. Section S4 in the Supplementary Material explains our technique to synchronize the Shimmer sensors with the already synchronized APDM sensors. By initializing all sensors through their respective systems and connecting them to a single PC, we aligned their internal clocks with the PC’s clock, minimizing the clock drift risk. Considering the brief duration of our data collection sessions, typically under one minute, the potential for significant clock drift was substantially reduced. Both the Falltrak II system and the IMU sensors were calibrated before each session.

### 2.5 Study design

Participants underwent a series of methodically structured steps as part of the study design.

• **Placements of wearables:** IMU sensors were placed on the participants’ bodies as described in Section 2.3
• **Positioning on Falltrak:** Participants stood barefoot on the Falltrak II platform, with specific instructions for initial positioning to ensure accuracy in balance measurement. Participants were instructed to adopt a standardized stance throughout the testing: feet shoulder-width apart, arms hanging at the sides, and eyes directed forward with no repositioning between conditions to maintain the continuity and efficiency of the test procedure. The procedure was immediately halted if any deviation from the prescribed posture was observed, such as unauthorized movement of the arms or opening of the eyes during conditions requiring them to be closed. Additionally, immediate support was provided to participants who showed signs of instability or were at risk of losing balance, thereby preserving the consistency of the test conditions and safeguarding participant well-being.
• **Order of measurements:** Tests conducted in a fixed sequence listed below. This order was designed to increase the challenge to participants’ balance systems progressively. In addition, these tests assess balance performance when one or more sensory systems are compromised.
  - **Eyes open, stable surface (EOSS):** Subjects stood on the hard surface of the platform with their eyes open. Participants stand on a stable surface with their eyes open in this condition. Here, all three primary sensory systems for balance (visual, somatosensory, and vestibular) are available for maintaining posture and equilibrium. The EOSS condition does not intentionally compromise any sensory system; instead, it serves as a baseline to evaluate balance under normal conditions where the visual and somatosensory inputs are intact and unaltered.
  - **Eyes closed, stable surface (ECSS):** Participants stood on the hard surface but with their eyes closed, increasing reliance on somatosensory and vestibular inputs for balance.
  - **Eyes open, foam surface (EOFS):** This condition involved standing on a foam pad placed on the platform with eyes open. This setup is designed to challenge the somatosensory system by introducing an unstable surface under the feet, compromising the reliability of somatosensory feedback used for balance. The visual and vestibular systems remain engaged and unaltered, providing the primary sources of sensory input for balance maintenance in this condition. The foam surface effectively simulates a compromised somatosensory condition, testing the participant’s ability to maintain balance with reduced somatosensory input.
  - **Eyes closed, foam surface (ECFS):** In the most challenging condition, subjects stood on the foam pad with their eyes closed, significantly reducing visual and somatosensory feedback.
• **Duration of each test and no breaks:** Each test condition lasted for approximately 11 s, following the 10 s guideline from the Falltrak II system to ensure compatibility with the equipment’s data collection parameters while still obtaining meaningful balance performance metrics (17). The tests were conducted without breaks between tests to simulate continuous balance challenges and streamline the assessment process.

### 2.6 Pre-processing considerations

Following data collection, we obtained one accelerometer and one gyroscope recording from each wearable sensor for all the experimental conditions: EOSS, ECSS, EOFS, and ECFS. Associated with each condition, we also derived the AV and PL scores from the Falltrak II system, representing the m-CTSIB scores. We used the chest-mounted microphone’s recorded vocal cues to determine the start and stop of each condition and segment each participant’s wearable sensor data into four distinct files, each corresponding to a different experimental condition. To account for potential transitions on or off the board, which could skew our analysis, we omitted a 0.5 s interval from the beginning and the end of each wearable sensor data segment. This pre-processing resulted in wearable data, with an average duration of 11 s (±1.6 s standard deviation) per condition. Accompanying each wearable sensor recording were their corresponding m-CTSIB AV and PL scores from the Falltrak II system. These data were then organized and stored in .csv format for further analysis.

In our study, among the participants, 31 were right-sided and 3 were left-sided. Recognizing the significant influence of limb dominance on postural stability and control, as highlighted in prior research by Promsri et al. and Yoshida et al., we categorized the sensor data to reflect each individual’s dominant and non-dominant sides (33, 34). This approach ensures a more accurate representation of balance performance, taking into account the variability introduced by side dominance.

Our decision to focus on estimating AV scores through our machine learning models is rooted in the clinical significance of AV in balance assessments for evaluating balance and stability, where higher scores indicate increased instability (35, 36). This emphasis on AV is further supported by our analysis, which revealed a strong Pearson correlation coefficient between AV and PL scores across all test conditions—0.94 for EOSS, and 1.00 for ECSS, EOFS, and ECFS. This high correlation demonstrates that variations in AV correspond closely with changes in PL, highlighting their interconnectedness in assessing balance.

We also decided to develop a single machine learning model for all EOSS, ECSS, EOFS, and ECFS experimental conditions. This approach improves the diversity of the dataset and the model’s ability to generalize, reflecting varied sensory and environmental challenges. Our analysis showed consistent sensor data patterns across conditions, supporting the effectiveness of one model to accurately estimate AV scores in diverse experimental conditions, enhancing both accuracy and versatility for balance assessment and rehabilitation applications.

### 2.7 Feature extraction

For each wearable sensor data, we extracted features independently from the *X*, *Y*, and *Z* axes. This process yielded a total of 42 features for each IMU data. Features were extracted from the full, unsegmented signal to maintain data integrity within each m-CTSIB test condition, as we chose not to implement signal segmentation due to the short duration of our data segments (approximately 11 s each). The extracted features (listed in Table 3) encompassed a wide range of data characteristics for their potential to reveal the subjects’ balance performance. These features are described in (37) and include:

• **Statistical features:** These features, such as standard deviation (SD), skewness, kurtosis, and sparsity, provide insights into the distribution and variability of the sensor signals and the stability and consistency of the subjects’ balance.
• **Time-domain features:** Features like difference sum, average jerk, and cross-correlation (*XY, YZ, XZ*) capture movement dynamics over time, reflecting how balance is maintained or adjusted.
• **Entropy measures:** Shannon entropy, sample entropy, and frequency-domain entropy offer an understanding of the complexity and predictability of the sensor signal patterns, which are indicative of balance control mechanisms.
• **Frequency-domain features:** These features, including the power of the main and secondary frequencies and their respective frequency values, reveal the dominant patterns of movement and rhythmic stability.

**Table 3:** The extracted features for each wearable sensor recording.

	Feature name	Signals	# Features
Statistical	1 – Standard deviation	X,Y,Z	3
	2 – Skewness	X,Y,Z	3
	3 – Kurtosis	X,Y,Z	3
	4 – Sparsity	X,Y,Z	3
Entropy	5 – Shannon entropy	X,Y,Z	3
	6 – Sample entropy	X,Y,Z	3
	7 – Frequency-domain entropy	X,Y,Z	3
Frequency	8 – Power of the main frequency	X,Y,Z	3
	9 – Power of the secondary frequency	X,Y,Z	3
	10 – Main frequency	X,Y,Z	3
	11 – Secondary frequency	X,Y,Z	3
Time	12 – Difference sum	X,Y,Z	3
	13 – Average jerk	X,Y,Z	3
	14 – Cross correlation XY	X,Y	1
	15 – Cross correlation XZ	X,Z	1
	16 – Cross correlation YZ	Y,Z	1
Total number of features			42

### 2.8 Machine learning models and feature selection

Predicting AV scores from wearable-derived features was formulated as a regression problem: AV=f(\,features), where ’features’ are derived from the wearable sensor data. In selecting the appropriate machine learning models for our study, we considered various factors, such as the nature of our data, the complexity of the regression problem, and the need for both interpretability and predictive accuracy. Multiple Linear Regression (MLR) was chosen for its simplicity and ease of interpretation, providing a clear understanding of how each feature influences the AV scores linearly. MLR models the relationship between a dependent variable Y (AV scores) and independent variables $X_{1},X_{2},\ldots ,X_{n}$ (wearable-derived features):

$$Y=\beta_{0}+\beta_{1}X_{1}+\beta_{2}X_{2}+\cdots +\beta_{n}X_{n}+\epsilon$$

Here, $\beta_{0}$ is the intercept, $\beta_{1},\beta_{2},\ldots ,\beta_{n}$ are the coefficients, and ϵ is the error term (38).

Support Vector Regression (SVR) was selected due to its effectiveness in handling non-linear relationships and its robustness to outliers, common in sensor data. SVR is a SVM variant used for regression problems (39). The SVR model can be represented as:

Y=⟨w,ϕ(X)⟩+b

where Y is the AV score, X is the feature vector, ϕ(X) is the feature vector transformed by the kernel function, w is the weight vector, and b is the bias. The kernel function transforms the original data into a higher dimensional space where a linear regression can be fit.

Finally, eXtreme Gradient Boosting (XGBOOST) was included for its advanced capabilities in handling complex, high-dimensional data and its inherent feature selection mechanism, making it adept at capturing intricate patterns in the data (40). The core principle of XGBOOST involves sequentially constructing an ensemble of decision trees, where each tree is built to correct the residuals or prediction errors made by the preceding trees. This additive model is represented as:

$$Y=\sum_{k=1}^{K}f_{k}(X),\;f_{k}\in F$$

where Y is the AV score for the feature set, X, K represents the number of boosting rounds (trees), and F is the space of all regression trees.

### 2.9 Training and testing setup

We applied both Subject-wise One-Leave-Out and 5-fold cross-validation methods for splitting the dataset into training and testing sets. For each iteration, one subject’s data was set aside for testing in the One-Leave-Out method, and for the 5-Fold method, data was divided into five parts, with one part used as the test set in each fold. To ensure the reliability and generalizability of our models, the training data was shuffled before being divided into training and validation sets, with 80% of the data used for training and the remaining 20% for validation. This validation set was used as an interim test to fine-tune model hyperparameters and avoid overfitting.

Hyperparameters for our models were optimized through a grid search strategy, focusing on the key parameters of each model. For SVR, we focused on optimizing the regularization parameter C between 0.1 and 10, epsilon ε from 0.01 to 0.2, and Linear and Radial Basis Function (RBF) kernel functions. In the case of XGBOOST, the feature subsampling rate range was set as (0.1,0.5). For maximum depth, we explored values from 3 to 10 in steps of 2 (i.e., 3 ≤ maximum depth ≤ 10, step=2), and for number of trees, the range was from 10 to 200 in increments of 20 (i.e., 10 ≤ number of trees ≤ 200, step=20).

The performance of our machine learning models was evaluated based on minimizing the Mean Absolute Error (MAE) between the predicted AV scores from the wearable sensor data and their ground truth AV scores from Falltrak II. We also provided the Pearson Correlation coefficient (r) as another objective evaluation metric.

#### 2.9.1 Feature selection strategy

During each iteration of the MLR and SVR subject-wise One-Leave-Out or 5-fold cross-validation, we calculated Pearson correlation coefficients between each IMU-derived feature and the AV scores from the training subset. Only features with a correlation coefficient above 0.7 were chosen for model inputs. This approach prevented leakage between the training and testing datasets and ensured the inclusion of features with a significant linear relationship with the AV scores. No separate feature selection was necessary for XGBOOST, which integrates its feature selection within the learning algorithm.

## 3 Results

This section presents a detailed analysis of the results obtained from our study. This includes FalltrakII measurement reports, a thorough analysis of features, an evaluation of the optimal sensor placement for estimating m-CTSIB AV scores, and a comprehensive assessment of the performance of our three machine learning methodologies.

### 3.1 Falltrak II measurements for participants

**Figure 3:**
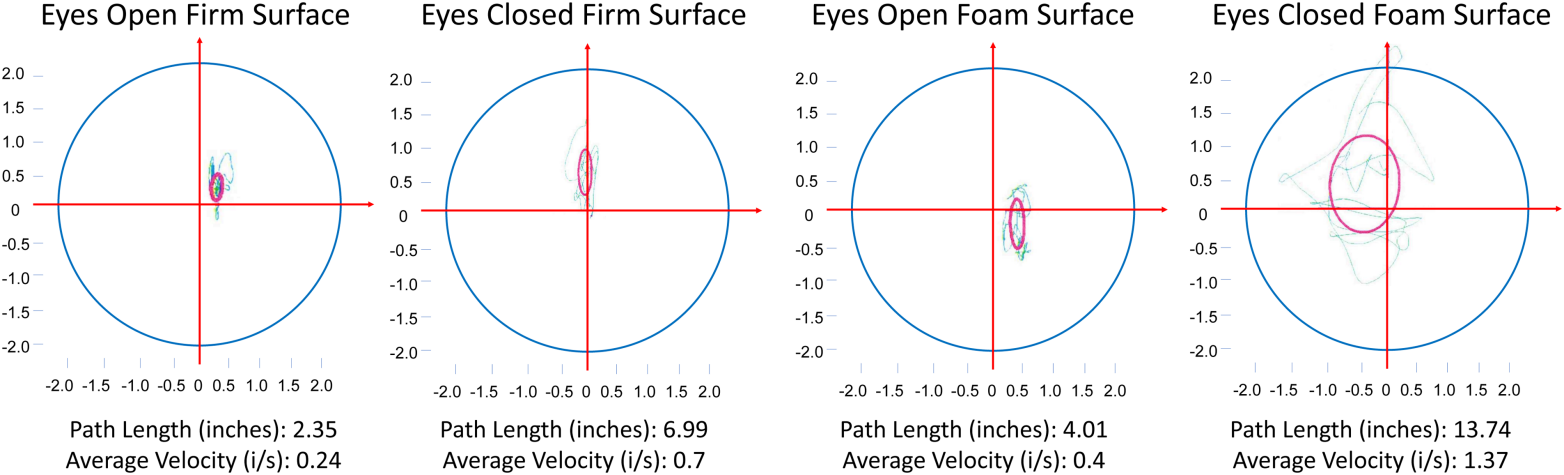
The variations in path length (PL) and average velocity (AV) across four m-CTSIB conditions of one of our subjects. The horizontal and vertical axes represent the displacement of the subject along the *x*-axis and *y*-axis, respectively. The green lines represent the real-time tracing of the subject’s center of pressure (COP) during the test, and the pink circles indicate the standard deviation of the subject’s COP.

**Figure 4:**
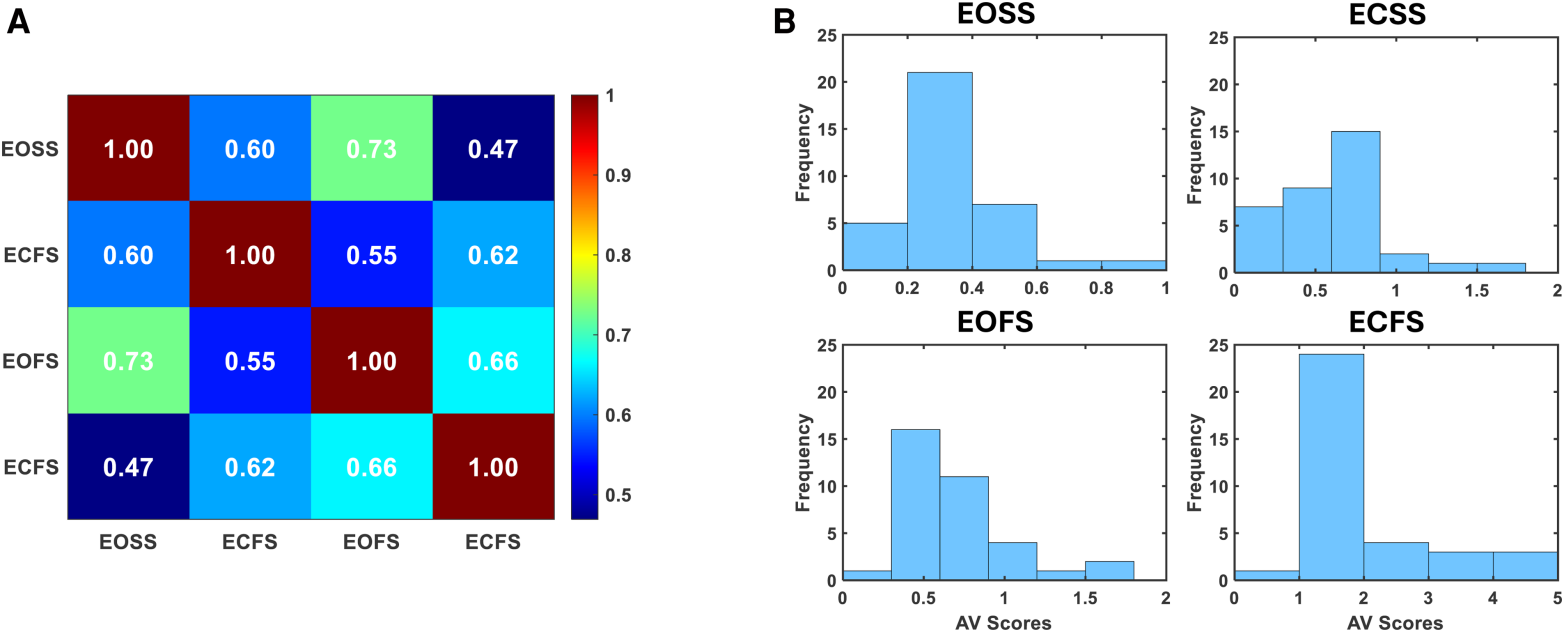
Falltrak II AV score analysis. (**A**) Depicts the correlation analysis of AV scores across four distinct m-CTSIB conditions. (**B**) Illustrates the histogram across four distinct m-CTSIB conditions.

Falltrak II traces participants’ real-time COP during the m-CTSIB test. Figure 3 shows the Falltrak II report for a participant and how PL and AV vary through different conditions, with ECFS being the most challenging with the highest PL and AV scores. Table 4 lists the mean and SD values of PL and AV for the study participants. PL is a measure of how much the COP moves during the test. A shorter path length indicates a better balance performance. AV is a measure of how fast the COP moves during the test. A lower average velocity indicates a better balance performance. We conducted a correlation analysis to evaluate the relationship between AV scores across EOSS, ECFS, EOFS, and ECFS conditions. Figure 4A illustrate a substantial correlation between the eyes-open conditions, EOSS and ECFS, with coefficients reaching 0.73. Conversely, the least challenging condition (EOSS) demonstrates the lowest correlation with the most demanding condition (ECFS), yielding coefficients of 0.47. Additionally, Figure 4B presents a histogram analysis comparing these conditions, revealing variations in the distribution of AV scores across them. This observation suggests that each condition poses a unique challenge for balance assessment, offering novel insights into the assessment of balance.

**Table 4:** Summary of average velocity (AV) and path length (PL) for test conditions.

	EOSS	ECSS	EOFS	ECFS
AV (inches/second)	0.33±0.16	0.63±0.32	0.70±0.36	1.94±1.00
PL (inches)	3.32±1.58	6.36±3.16	7.05±3.58	19.47±9.99

Values are presented as mean ± SD. AV and PL stand for average velocity and path length from the COP, respectively. AV, average velocity; PL, path length; EOSS, eyes open, stable surface; ECSS, eyes closed, stable surface; EOFS, eyes open, foam surface; ECFS, eyes closed, foam surface.

### 3.2 Feature analysis of wearable sensors

**Figure 5:**
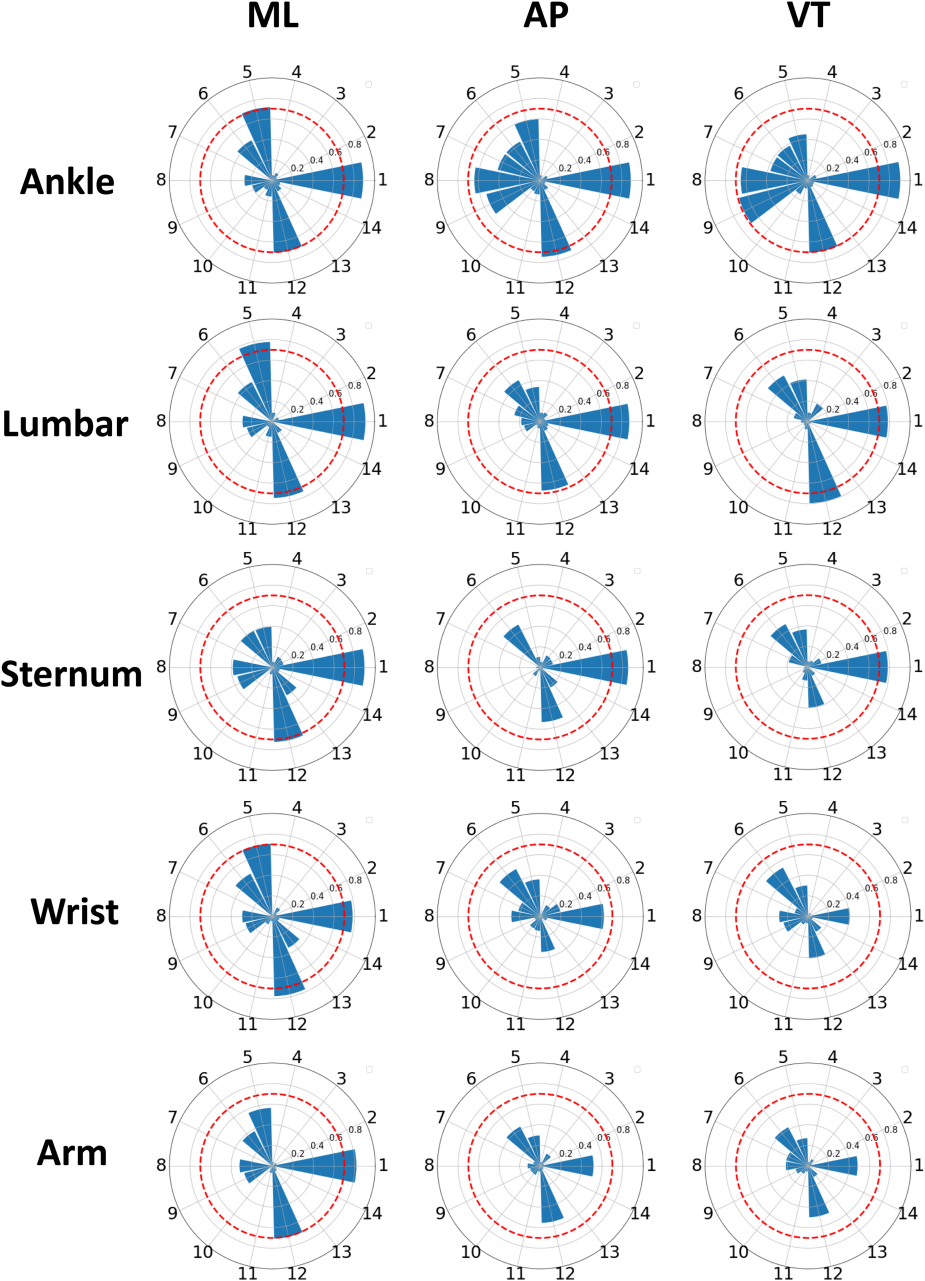
Radar plots of feature correlations across sensors. Radar plots illustrate the correlation of accelerometer-derived features from the medial-lateral (ML), anterior-posterior (AP), and vertical (VT) axes with average velocity (AV) balance scores. Features are represented on spokes with correlation coefficients ranging from 0 to 1. The red radar indicates a correlation of 0.7, and features with correlations exceeding this threshold are considered significant.

We extracted features from the accelerometer signals collected during each m-CTSIB condition as explained in Section 2.7. Our feature analysis was conducted to determine the relevance of these sensor-derived features in predicting m-CTSIB AV scores. To ensure uniform contribution across all features in our model, each was normalized using its mean and standard deviation. This normalization process prevented any feature from dominating due to scale variance. We then computed correlation coefficients between the normalized features and the AV scores to assess the relevance of each feature to balance. Figure 5 displays a series of radar plots for different sensor locations: the ankle, lumbar, sternum, wrist, and arm. These plots illustrate the correlation coefficients of each feature from 0 to 1, with higher radial distances indicating stronger correlations. The features arranged counterclockwise as per Table 3, include cross-correlation features (*XY*, *XZ*, and *YZ*) as the 14th feature on respective axes. The analysis revealed that features related to ML movements showed the highest correlation values, followed by those related to AP movements, highlighting the significance of ML and AP movements in balance control. Among the sensors, the ankle showed the highest correlation values for balance-related features, followed by lumbar, sternum, wrist, and arm sensors in that order.

**Figure 6:**
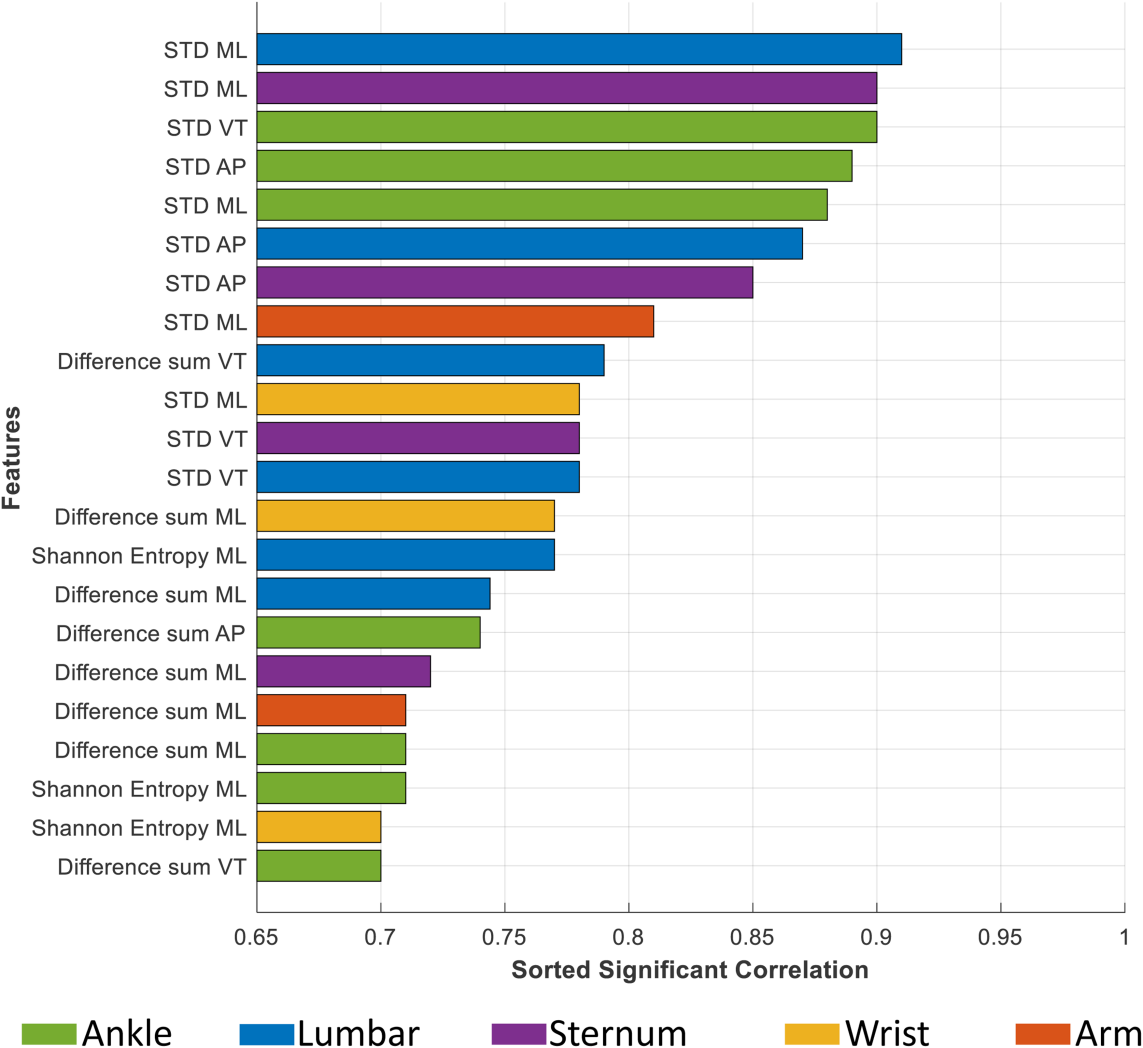
This figure illustrates the significant features of each sensor along three axes: medial-lateral (ML), anterior-posterior (AP), and vertical (VT), identified by a correlation coefficient exceeding 0.7. The colors represent different sensor placements.

**Figure 7:**
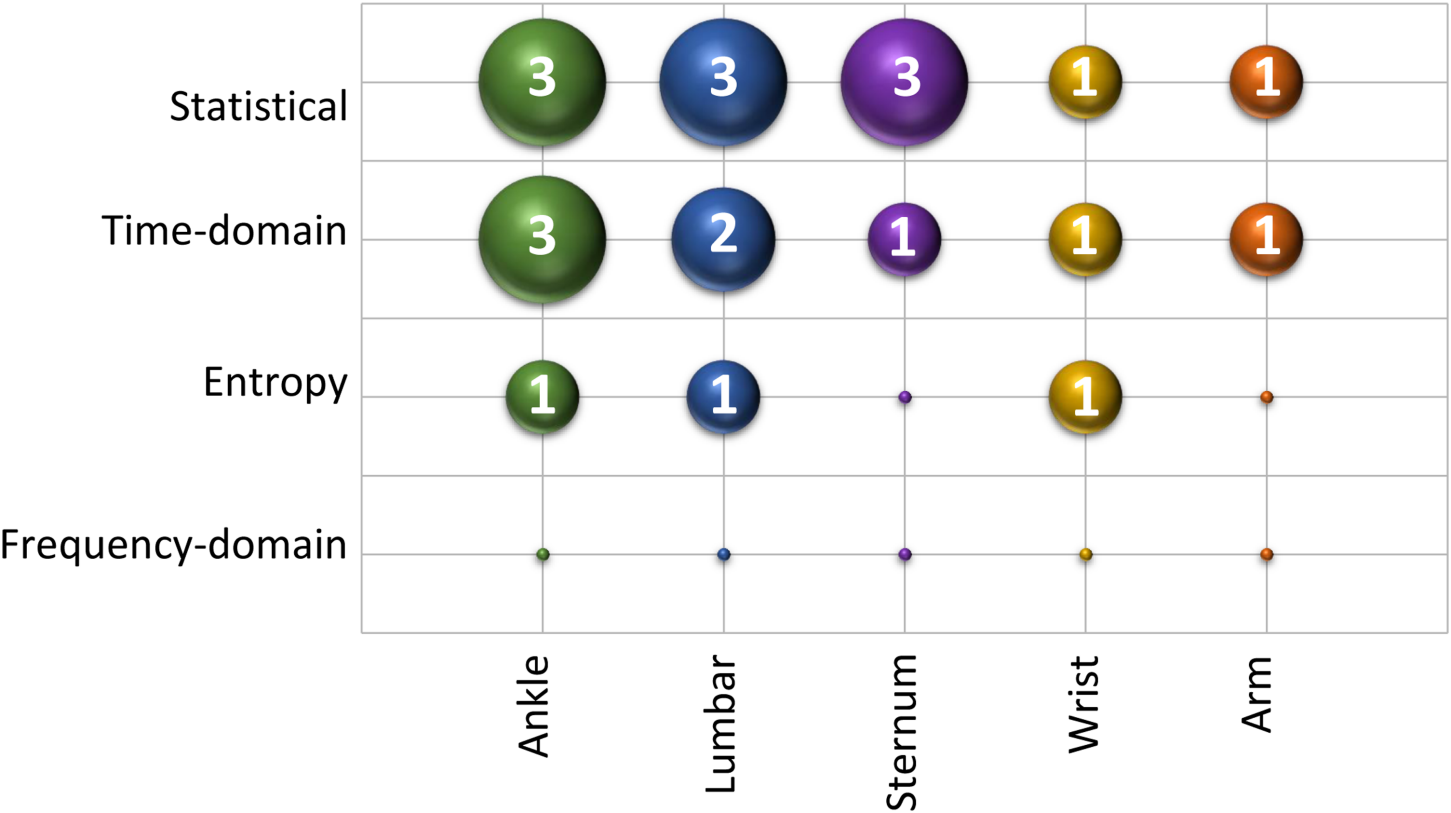
Distribution of significant features by sensor placement and feature type.

We considered features with a correlation coefficient greater than 0.7 with the balance score as significant features. Figure 6 showcases these significant features for each sensor location. This figure reveals a notable presence of features related to variability metrics, such as STD and difference sum, as well as entropy-based features. Their dominance implies that sensor-captured movement variations are critical in indicating balance stability or instability, with higher STD values, for example, potentially reflecting greater instability. Figure 7 offers an insight into the distribution of these significant features across sensor locations and feature types. It shows that the ankle and lumbar sensors have the most substantial number of significant features, with 7 and 6 features, respectively. These locations represent 32% and 27% of all significant features identified, highlighting their importance in accurately estimating m-CTSIB AV scores. The graph also emphasizes the prevalence of statistical and time-domain features as key predictors of balance while noting the absence of significant frequency-domain features in any sensor placements. This distribution underscores the relevance of specific feature types and sensor locations in balance assessment and aids in optimizing the balance evaluation process.

### 3.3 m-CTSIB score estimation

Our experiments employed specific Python packages: sklearn.linear_model for implementing MLR, sklearn.svm for SVR, and the xgboost package for implementing the XGBOOST algorithm. We followed the training and testing setup described in Section 2.9 and reported the optimal hyperparameters for the SVR and XGBOOST models of each sensor placement in Table 5. We observed variability in hyperparameter values across different cross-validation folds, stemming from each fold featuring a distinct training and validation data combination. This diversity necessitates adjustments in model parameters to best fit each specific data distribution. Moreover, the range of optimized hyperparameters varied between the One-Leave-Out and 5-Fold cross-validation methods. The One-Leave-Out approach, with its detailed analysis per fold, permits a wider exploration of hyperparameter settings. In contrast, the 5-fold method consolidates findings across multiple folds, requiring a more cautious hyperparameter selection to maintain model generalizability while avoiding overcomplexity.

**Table 5:** Optimized hyperparameters for best models across various sensor locations.

	Sensors	SVR			XGBOOST		
		Kernel	C	ϵ	Number of trees	Maximum depth	Feature subsampling rate
One-Leave-Out	Ankle	Linear	[0.1–10]	[0.01–0.2]	[30–190]	[3–9]	[0.1–0.4]
	Lumbar	Linear	[0.1–10]	[0.01–0.2]	[30–190]	[3–9]	[0.1–0.4]
	Sternum	Linear	[0.1–10]	[0.01–0.2]	[30–190]	[3–9]	[0.1–0.4]
	Wrist	RBF	[0.1–10]	[0.01–0.2]	[50–190]	[3–9]	[0.1–0.4]
	Arm	Linear	[0.1–10]	[0.01–0.2]	[30–190]	[3–9]	[0.2–0.4]
5-Fold	Ankle	Linear	[0.1–1]	[0.01–0.2]	[30–170]	[3–9]	[0.1–0.4]
	Lumbar	Linear	[0.1–1]	[0.1–0.2]	[30–150]	[5–6]	[0.3–0.4]
	Sternum	Linear	[0.1–1]	[0.1–0.2]	[30–190]	[3–9]	[0.2–0.4]
	Wrist	RBF	[1–10]	[0.01–0.1]	[70–110]	[3–9]	[0.1–0.4]
	Arm	Linear	[1–10]	[0.01–0.1]	[30–130]	[3–9]	[0.3–0.4]

Values are presented as [range].

The validation and test results, including r and MAE, are detailed in Table 6. Specifically, with One-Leave-Out cross-validation, lumbar sensor results showed r of 0.92 and MAE of 0.23 using MLR. The same sensor achieved r values of 0.90 and 0.96, and MAE values of 0.24 and 0.23 with SVR and XGBOOST, respectively. In 5-fold cross-validation, the lumbar sensor’s performance included r values of 0.55 to 0.92 and MAE from 0.50 to 0.23 across MLR, SVR, and XGBOOST.

**Table 6:** Subject-wise One-Leave-Out and 5-fold cross-validation performance using various sensor placements and machine learning models.

Methods	Sensors	One-Leave-Out				5 Fold			
		Validation		Test		Validation		Test	
		MAE ± SD	r	MAE ± SD	r	MAE ± SD	r	MAE ± SD	r
MLR	Ankle	0.27±0.05	0.90	0.25 ± 0.21	0.91	0.29±0.13	0.90	0.51 ± 0.07	0.58
	Lumbar	0.23±0.06	0.94	**0.23 ± 0.15**	**0.92**	0.28±0.13	0.94	**0.50 ± 0.06**	**0.55**
	Sternum	0.28±0.06	0.91	0.29±0.20	0.88	0.30±0.07	0.94	0.51±0.07	0.56
	Wrist	0.43±0.12	0.77	0.40±0.33	0.71	0.54±0.13	0.78	0.49±0.08	0.54
	Arm	0.42±0.15	0.78	0.38±0.36	0.75	0.36±0.18	0.76	0.52±0.07	0.55
SVR	Ankle	0.21±0.07	0.91	0.27 ± 0.22	0.88	0.40±0.07	0.93	0.34 ± 0.05	0.84
	Lumbar	0.25±0.06	0.93	**0.24 ± 0.18**	**0.90**	0.45±0.12	0.89	**0.31 ± 0.03**	**0.85**
	Sternum	0.29±0.07	0.88	0.30±0.20	0.87	0.33±0.02	0.85	0.39±0.10	0.86
	Wrist	0.38±0.19	0.81	0.31±0.20	0.84	0.36±0.06	0.80	0.47±0.12	0.68
	Arm	0.33±0.09	0.78	0.34±0.29	0.77	0.44±0.08	0.62	0.47±0.10	0.63
XGBOOST	Ankle	0.18±0.02	0.91	0.26 ± 0.15	0.94	0.19±0.02	0.91	0.27 ± 0.04	0.89
	Lumbar	0.15±0.02	0.95	**0.23 ± 0.15**	**0.96**	0.13±0.01	0.97	**0.23 ± 0.03**	**0.92**
	Sternum	0.20±0.02	0.90	0.30±0.15	0.88	0.20±0.02	0.85	0.29±0.06	0.89
	Wrist	0.20±0.03	0.92	0.32±0.20	0.90	0.19±0.05	0.92	0.33±0.09	0.81
	Arm	0.20±0.03	0.93	0.30±0.24	0.88	0.19±0.02	0.95	0.35±0.12	0.71

The bold values represent the optimal outcomes achieved by machine learning algorithms, as determined through both One-leave-out and 5-fold cross validation.

Similarly, the ankle sensor demonstrated strong performance. During One-Leave-Out cross-validation, it reached r of 0.91 and MAE of 0.25 with MLR, and for SVR and XGBOOST, it recorded r values of 0.88 and 0.94, and MAE values of 0.27 and 0.26, respectively. The 5-fold cross-validation for the ankle sensor showed r ranging from 0.58 to 0.89 and MAE from 0.51 to 0.27 across the three machine learning models.

In refining our models, we addressed feature redundancy by excluding highly correlated features with a correlation of >0.9 with each other. This adjustment aimed to streamline the feature set for MLR and SVR algorithms. Our observations suggested a negligible effect on model efficacy, with a slight performance decrease in certain cases. This outcome implied that given the modest size of the initial significant feature set (up to seven features), even redundant features could be instrumental in our model’s prediction capacity. Therefore, while minimizing redundancy is a standard practice to avert model bias, our analysis showed that preserving these features could be beneficial for maintaining the predictive strength of the models.

Moreover, we explored the potential of incorporating gyroscope data instead of accelerometer data, given that our IMU sensors capture both types of measurements. Section S5 in the Supplementary Material provides a detailed analysis. The analysis, however, affirmed the superior performance of accelerometer data in terms of correlation with actual balance scores and lower MAE, leading us to prioritize accelerometer data in our primary analysis. The preference for accelerometer data is further supported by advantages such as lower power consumption, cost-effectiveness, and broader accessibility, making them a preferable option for continuous health monitoring.

We repeated our investigation using the lumbar and ankle sensors for the One-Leave-Out method. However, combining lumbar and ankle sensors did not enhance performance as expected, resulting in a lower testing r of 0.62 and MAE of 0.43 for MLR, 0.62 and 0.42 for SVR, and 0.95 and 0.27 for XGBOOST. This could be due to the increased complexity and potential redundancy in the data when combining sensors, which might not linearly translate to improved predictive accuracy.

Furthermore, we computed the MAE for each condition individually from the lumbar sensor data. The findings indicate that within eyes-open conditions (i.e., EOSS and EOFS), the MLR model achieved the lowest MAE of 0.17 vs. an MAE of 0.20 obtained using XGBOOST. Conversely, during the eyes closed conditions (i.e., ECSS and ECFS), the XGBOOST model exhibited the minimum MAE of 0.26 vs. 0.29 using the MLR model. Such a difference in performance could be attributed to the nature of the data and the models’ strengths. MLR, being a linear model, may perform better when the relationship between the input features and the output is more linear, which might be the case in eyes-open conditions. In contrast, XGBOOST, a more complex and non-linear model, could better capture the subtler, more complex patterns in the eyes-closed conditions, where maintaining balance might depend on less obvious or non-linear relationships in the data. The closed-eye conditions likely introduce more variability and complexity in the balance data, which non-linear models like XGBOOST are better equipped to handle.

**Figure 8:**
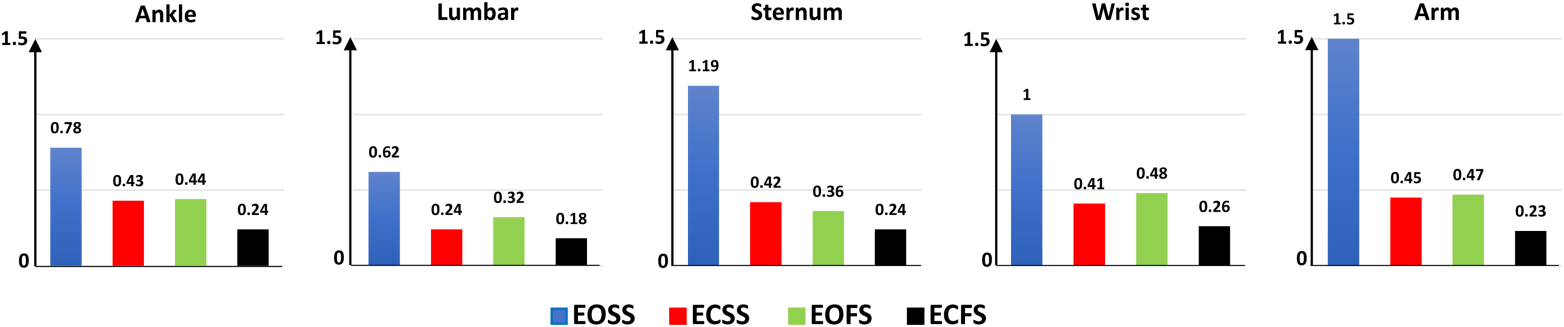
Mean absolute percentage error (MAPE) of the XGBOOST model for m-CTSIB conditions—EOSS, eyes open on solid surface; ECSS, eyes closed on solid surface; EOFS, eyes open on foam surface; ECFS, eyes closed on foam surface.

Figure 8 presents the Mean Absolute Percentage Error (MAPE) for the XGBOOST model in the One-Leave-Out method across different conditions—EOSS, ECSS, EOFS, and ECFS—for sensor locations including ankle, lumbar, sternum, wrist, and arm. MAPE was chosen as the evaluation metric over MAE due to the varying ranges of balance scores among these conditions, facilitating more effective result comparisons, as detailed in Table 4. ECFS, EOFS, and ECSS conditions show notably low MAPE values, with ECFS achieving the lowest MAPE in the range of 0.18–0.26. This suggests the models excel in predicting balance scores under these challenging conditions, showcasing their proficiency in scenarios where maintaining balance is considerably more difficult.

**Figure 9:**
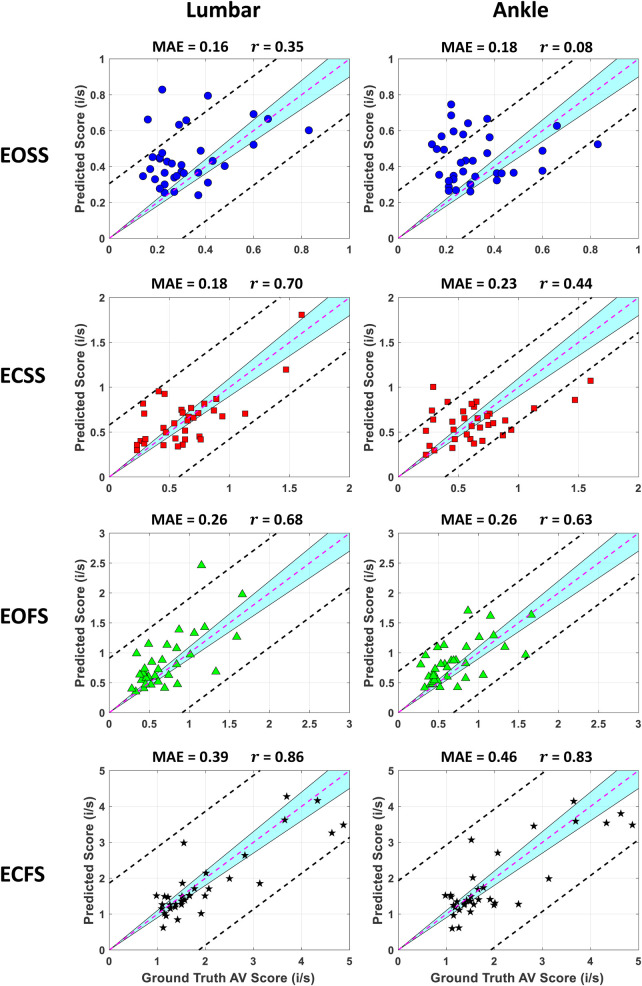
Scatter plots comparing predicted AV scores of the XGBoost method from wearable sensor data to ground truth AV scores across multiple m-CTSIB conditions. Each plot corresponds to a different condition, with data points color-coded for clarity. The dashed lines indicate the bounds of a 95% confidence interval. The abbreviation i/s indicates inches/second.

Figure 9 illustrates the correlation between ground truth and predicted AV scores for EOSS, ECSS, EOFS, and ECFS from the XGBOOST One-Leave-Out methods applied to lumbar and ankle sensor data. The plot reveals a high concentration of predictions, marked by color-coded data points with distinct markers, aligning closely with the r=1 line, depicted as a purple dashed line. This pattern suggests that the models demonstrate robust performance in the AV score prediction. Notably, predictions from the lumbar sensor placement are generally superior to those from the ankle, as evidenced by the data points’ proximity to the 95% prediction band (indicated by the black dashed lines), being more distant in the case of the lumbar.

**Figure 10:**
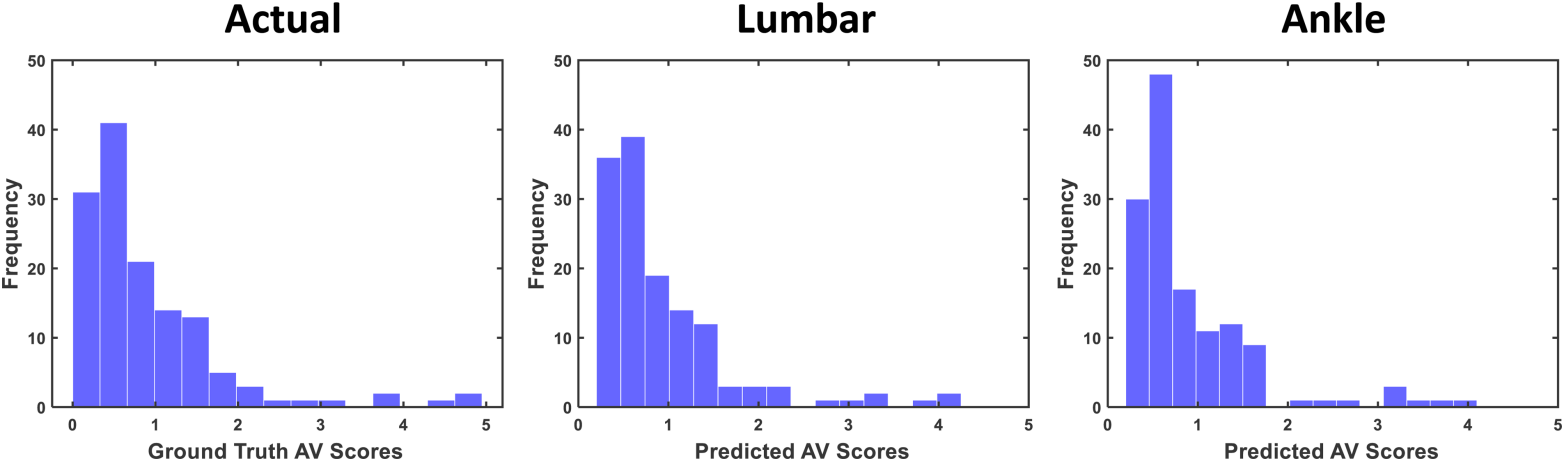
Distribution of actual vs. predicted m-CTSIB scores of the XGBOOST from One-Leave-Out cross validation.

To visualize the distribution of both actual m-CTSIB scores and predicted scores from our model, refer to Figure 10. This figure presents histograms comparing the ground truth m-CTSIB scores with the scores predicted by our XGBOOST model using the One-Leave-Out method for the ankle and lumbar sensors. The alignment between these two distribution sets underscores the predictive accuracy of our model across various conditions.

## 4 Discussion

Our study investigated wearable sensors for objective m-CTSIB balance score estimation under various sensory conditions defined by the test. This objective emerged from recognizing the need for advanced tools to capture the nuanced effects of different sensory inputs on balance. Traditional balance assessments often lack the granularity to dissect these influences comprehensively, leading to a gap in our understanding and management of balance impairments. Moreover, wearables support remote monitoring, enabling healthcare professionals to evaluate patients’ balance remotely, which is particularly useful in diverse healthcare scenarios (41, 42).

### 4.1 Key findings and observations

Our main finding was that wearable sensors combined with machine learning could effectively estimate AV scores during m-CTSIB tests. The most notable performance was achieved using data from the lumbar sensor with the XGBOOST method, resulting in a low MAE of 0.23 using One-Leave-Out and 5-fold cross-validation and a high correlation of 0.96 and 0.92 using One-Leave-Out and 5-fold cross-validation, respectively (Table 6). However, when considering specific scenarios, we found that MLR was more suitable for eyes-open conditions, while XGBOOST was better suited for eyes-closed conditions. This distinction suggests the benefit of employing different models tailored to the specific sensory conditions of the m-CTSIB test, optimizing the balance assessment’s accuracy and reliability. Despite the promising results, our study also acknowledged limitations, particularly the higher MAPE observed in simpler tasks like the EOSS condition (Figure 8). This was attributed to the low base values of m-CTSIB scores in these tasks, where small predictive errors could disproportionately inflate the error percentage. However, this limitation does not detract from the utility of our models in more complex conditions, which are of greater clinical interest for identifying balance impairments related to cognitive decline or neurological conditions.

Another main observation was that the lumbar and dominant ankle sensors were the most effective in estimating m-CTSIB balance scores. In contrast, dominant arm and wrist sensors were the least effective (Table 6). This pattern reflects the biomechanical realities of balance control. Lumbar and ankle regions are central to maintaining postural stability, directly influencing the body’s center of gravity and subtle balance adjustments, which aligns with prior studies (43, 44). In contrast, the arm and wrist play a more secondary role in overall balance, contributing less to core postural stability. This highlights the importance of sensor placement in areas most integral to balance for more accurate and reliable assessments. Interestingly, combining data from the lumbar and ankle sensors did not enhance performance. Besides the practicality concerns of requiring two sensors for assessment, this outcome suggests that a single, well-placed sensor might be more efficient for balance evaluation.

Our feature analysis emphasized that movement variability significantly impacts balance performance. Specifically, a higher standard deviation indicates increased instability, marking it as a critical factor across all sensor placements (Figure 6). Moreover, the temporal characteristics of movement, including transition smoothness and body part coordination, play essential roles in balance control. While our analysis did not identify significant frequency-domain features due to predominant stable and consistent movement patterns within various frequency bands, it is critical to acknowledge that dynamic balance assessment involving activities like walking or stepping might necessitate incorporating frequency-domain features for a thorough analysis (45). We also found that the ML movements strongly correlated with m-CTSIB AV scores. This aligns with existing research, which suggests that balance adjustments primarily involve ML movements (46). This finding underscores the importance of these directional movements in maintaining and assessing balance, providing critical insights into postural control dynamics.

### 4.2 Comparative literature review

Table 7 provides an overview of prior research endeavors using wearable sensors and machine learning methodologies to estimate balance test outcomes. As depicted in Table 7, variations exist in sensor placement, machine learning models employed, participant numbers, and the most noteworthy outcomes achieved in each study. Despite its importance (21, 22), our study represents the first to estimate m-CTSIB AV scores objectively using wearable sensors and machine learning, distinguishing it from previous research that primarily focused on the BBS and one instance on the TUG test. Our participant number is comparable to other studies, reinforcing the validity of our findings. Unique to our approach was the exploration of five different sensor placements, with a detailed report on the most effective single placement, unlike other studies that did not conduct as extensive a placement analysis.

**Table 7:** Comparison of our study with previous studies.

Study	Balance test	# Participants	Best sensor placement (#)	ML model	Best results
Similä et al. (47)	BBS	49	Lumbar (1)	KNN	MAE=3.53
Shahzad et al. (48)	BBS	23	Lumbar (1)	LLS Lasso	MAE=1.44 r=0.90
Tang et al. (27)	BBS	30	Hip and foot (3)	SVR	MAE=6.07
Choi et al. (26)	TUG	37	Foot (2)	RR	MAE=0.87
Our study	m-CTSIB	34	Lumbar (1)	MLR SVR XGBOOST	MAE=0.23 r=0.96

ML, machine learning; KNN, k-nearest-neighbors; BBS, Berg balance scale; TUG, timed up and go; m-CTSIB, modified clinical test for sensory interaction and balance; SVR, support vector regression; LLS, linear least squar regression; RR, ridge regression.

### 4.3 Clinical implications and biomechanical insights

The clinical implications of our study are significant, offering a new, objective approach to balance assessment using wearable sensors and machine learning. By not depending on specialized equipment, such technology promises enhanced practicality for a broad audience, including older adults and those with mobility challenges. Additionally, it enables healthcare professionals to evaluate remote balance, opening new possibilities in various healthcare contexts. (41, 42).

Our findings reveal a significant reliance on ankle strategies for managing minor balance disturbances, a correlation that is particularly strong at the ankle sensors. This observation is in harmony with the work of Horak (49) and Nashner (50), who have documented the preference for ankle strategies when dealing with small shifts on a stable platform, utilizing the distal muscles for effective postural control. Nashner’s further discussions highlight the activation of hip strategies in response to larger balance disruptions, indicating a sophisticated balance control system that adapts based on the scale of the challenge.

Additionally, the effectiveness of the ankle musculature in maintaining balance with minimal energy and swift responsiveness is especially relevant for those with balance disorders, such as Parkinson’s disease (51, 52). This underscores the importance of considering both the nature of the perturbation and the individual’s physiological state when selecting balance strategies. These insights are crucial for devising targeted balance assessments and rehabilitation programs, affirming the value of our study in enhancing the understanding and treatment of balance impairments through tailored interventions.

### 4.4 Study limitations and future work

While our study has successfully demonstrated the potential of wearable sensors and machine learning in balance assessment, it has also highlighted areas for future enhancement. The sample size, though adequate for initial exploration, was limited, and a gender and hand dominance imbalance was noted, which may affect the representativeness of the results.

In addressing the complexities of upper limb movements within our study, we implemented rigorous Falltrak II data collection protocols to standardize participant posture and minimize potential variations. Despite these measures, the unique challenges posed by the degrees of freedom in arm movements remained. Future investigations could benefit from a more diverse and larger cohort to validate and extend our findings. Furthermore, to ensure our models’ resilience against varied movement patterns, we plan to test them rigorously with different types of motion distortions. This will help refine the models to be more adaptable and reliable across a wider spectrum of real-world scenarios.

We also acknowledge that the proprietary nature of normative databases used in commercial systems restricts the direct comparison between raw AV and PL scores and established stability scores. Moving forward, rather than relying on partnerships with external platforms or proprietary normative data, we will focus on leveraging our models’ raw AV and PL data. This approach will allow us to create a more precise and transparent framework for balance assessment. Specifically, we aim to utilize these predicted balance scores as foundational data for developing predictive models tailored to various applications, such as early detection of cognitive impairments or Alzheimer’s disease. Through this refined focus, our research is poised to make a meaningful contribution to advancing the field, offering novel insights and tools for the early identification and intervention of balance-related health issues.

## 5 Conclusion

Our study introduced a new method for accurately estimating AV scores during m-CTSIB balance tests, employing wearable sensors and machine learning techniques. By gathering detailed motion data from 34 participants under four distinct sensory conditions, we applied MLR, SVR, and XGBOOST machine learning models on a comprehensive subset of features derived from the wearable data to estimate their corresponding ground truth m-CTSIB AV scores. Our findings underscored our approach’s high accuracy and strong correlation with ground truth AV balance scores, particularly highlighting the exceptional performance of the XGBOOST model. Data from lumbar and dominant ankle sensors demonstrated the highest performance in balance score estimation, highlighting the importance of strategic sensor placement for capturing relevant balance adjustments and movements. Our findings pave the way for more precise and convenient balance assessments. This approach has immense potential to enhance balance performance assessment and management in various settings, including clinical environments, rehabilitation, and remote monitoring, offering a significant advancement in healthcare.

## Data Availability

Data supporting the findings of this study are available from the corresponding author on reasonable request.
