# Supplementary material for: Objective estimation of m-CTSIB balance test scores using wearable sensors and machine learning
Source: Front Digit Health. 2024 Apr 19;6:1366176. doi: 10.3389/fdgth.2024.1366176 (PMC11066210; doi:10.3389/fdgth.2024.1366176)
Supplement: Supplementary file 1 [file Datasheet1.pdf]

Supplementary Material

1 DETAILED SCORING METHODS FOR THE M-CTSIB

Clinical Scoring of m-CTSIB: The m-CTSIB simplifies the original CTSIB by eliminating the visual conflict dome, focusing on four key conditions to assess balance Cohen et al. (1993). Patients are evaluated while standing on both firm and compliant (foam) surfaces, with eyes both open and closed, across these conditions. Performance is timed for 30 seconds, with additional attempts allowed if the initial stance cannot be maintained, and the average of the trials is calculated for each condition. Importantly, research indicates that factors such as foot position or footwear do not significantly influence test scores Whitney and Wrisley (2004) Wrisley and Whitney (2004). A qualitative assessment of sway is also recommended, ranging from minimal (1) to a fall (4), providing a comprehensive measure of balance ability. This clinical approach to scoring is supported by studies underscoring the m-CTSIB's utility in predicting falls and assessing balance across various populations Boulgarides et al. (2003) Horak (1987) Park et al. (2013).

Instrumented Scoring with FallTrak II: For a more objective quantification of m-CTSIB scores, the FallTrak II system is utilized, incorporating a normative database from tests conducted on individuals aged 12 to 88 years, stratified into ten-year increments Freeman et al. (2018). This system enables a detailed comparative analysis through its Group Summary program, comparing individual test results against age-matched normative data. Such an instrumented approach enhances the interpretative capabilities of the m-CTSIB, offering a standardized method for assessing balance impairments with greater precision. The integration of FallTrak II's objective data analysis with clinical assessment methods provides a comprehensive framework for understanding and evaluating balance performance in clinical settings.

2 TECHNICAL SPECIFICATIONS AND COMPARATIVE ANALYSIS OF WEARABLE SENSORS

The technical specifications of both sensors are closely aligned, ensuring consistency in the data collected for our study's objectives. Table 2 in the manuscript presents a side-by-side comparison of the technical specifications for the APDM and Shimmer sensors. Both sensor types operate at a sampling rate of 128 Hz, cover a range of ± 16 g, and measure across three axes, providing a robust foundation for comparative analysis. While there is a minor difference in resolution—APDM sensors have a slightly higher resolution of 17.5 bits compared to the 16 bits of Shimmer sensors—the impact on data quality, especially for arm movement data, is minimal and within acceptable margins for our analysis.

3 INTERPRETING AV AND PL BALANCE METRICS

Our study incorporates AV and PL metrics within the framework provided by the FallTrak II system. This system is underpinned by a normative database derived from a wide age range of individuals (12 to 88 years), stratified into ten-year increments, enabling a nuanced comparative analysis of balance performance. AV and PL evaluate an individual's ability to maintain balance and postural stability. In clinical settings, AV and PL are indeed often correlated, as both are influenced by the individual's ability to maintain a stable posture. High values in these metrics typically indicate greater postural sway and potentially less stable balance, whereas lower values suggest more stable postural control. Specifically:

- AV reflects the speed of the Center of Pressure (COP) movement. A higher AV suggests quicker COP movements, indicating less stable balance.
- PL denotes the total distance traveled by the COP. A longer PL signifies more extensive COP movement, potentially reflecting greater effort or difficulty in maintaining balance.

Normative ranges for AV and PL vary across different populations and depend on age, physical condition, and specific clinical criteria. The FallTrak II system facilitates the interpretation of these metrics by comparing individual scores against age-matched normative data, categorizing performance as abnormal, borderline, or normal. This comparative analysis is crucial for identifying deviations from expected balance performance guiding clinical decision-making. Table S1 presents an example report of a study participant with AV and PL units in various conditions. Figure S1 illustrates the FallTrak II-reported stability scores for the participant referenced in Table S1.

Table S1. AV and PL scores for one study participant.

	**EOSS**	**ECSS**	**EOFS**	**ECFS**
**AV (inches/second)**	0.21	0.63	0.66	1.27
**PL (inches)**	2.14	6.34	6.62	12.67

AV and PL stand for average velocity and path length from the COP, respectively. *AV* = average velocity; *PL* = path length; *EOSS* = eyes open, stable surface; *ECSS* = eyes closed, stable surface; *EOFS* = eyes open, foam surface; *ECFS* = eyes closed, foam surface.

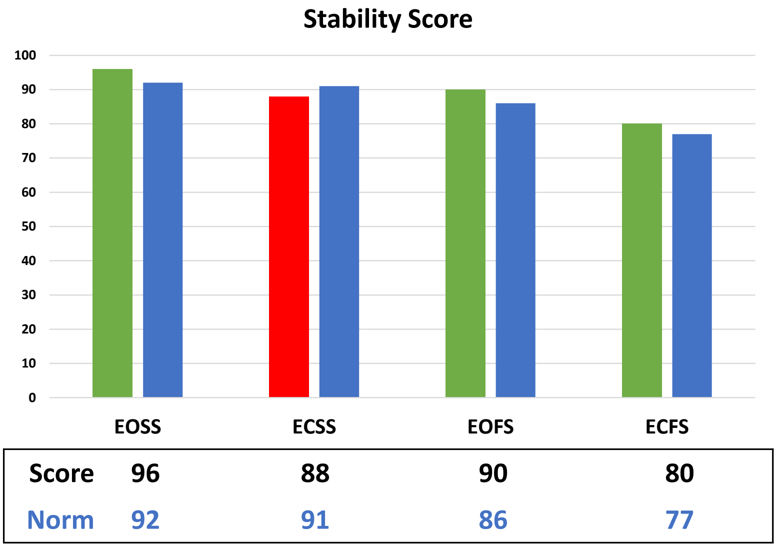

Figure S1. The stability scores for the participant reported in Table S1. The blue bars show the normative value for patient age range.

4 COMPARATIVE ANALYSIS OF ACCELEROMETER AND GYROSCOPE DATA

Our wearable sensors are equipped with both accelerometers and gyroscopes, and we have thoroughly examined data from both to estimate balance scores. The analysis, incorporating machine learning,

consistently demonstrated a preference for accelerometer data due to its stronger correlations and lower MAE between actual and predicted AV scores, compared to gyroscope data. Please see Table S2 presenting the results of both accelerometer and gyroscope sensor data from the XGBOOST algorithm. As can be seen the performance between the two types of sensors across various body locations is comparable, with accelerometers generally slightly outperforming. Furthermore, the choice to prioritize accelerometer data over gyroscope data in our main analysis is supported by the accelerometer's lower power consumption, greater affordability, and wider availability. These factors make accelerometers a more practical choice for continuous monitoring in wearable health devices.

5 SYNCHRONIZATION PROCESS FOR WEARABLE SENSORS

The APDM sensors underwent synchronization during their calibration phase, and a method was applied to synchronize the Shimmer sensors with the already synchronized APDM sensors. This approach involved executing a distinct physical motion—rapidly shaking the sensors up and down—to generate a recognizable pattern across all sensors. A MATLAB script was employed to detect the peak of the shaking pattern at the initiation of each sensor's recording. This peak served as a reference point for aligning the time axes of all sensors, with specific attention to ensuring accurate synchronization of the Shimmer sensors' data streams with the APDM sensors' time axis, established as the standard reference. Refer to Figure S2 in the supplementary material to illustrate this shaking process. In Figure S2A, the pre-synchronization data displays the specific pattern occurring at different times (indicated by red lines). These distinctive patterns in the sensor signals were utilized as annotations to synchronize all Shimmer and APDM sensor data. Figure S2B depicts the post-synchronization signals, with the conclusion of the recognizable patterns serving as the aligned point for all signals.

6 COMPARING THE CORRELATION BETWEEN GROUND TRUTH AND PREDICTED AV SCORED FOR DIFFERENT CONDITIONS

Figure S3 illustrates the correlation between ground truth and predicted AV scores for EOSS, ECSS, EOFS, and ECFS from the three machine learning methods using One-Leave-Out cross validation applied to lumbar and ankle sensor data. The plot reveals a high concentration of predictions, marked by color-coded data points with distinct markers, aligning closely with the $r = 1$ line, depicted as a purple dashed line. This pattern suggests that the models demonstrate robust performance in the AV score prediction. Notably, predictions from the lumbar sensor placement are generally superior to those from the ankle, as evidenced by the data points' proximity to the 95% prediction band (indicated by the black dashed lines), being more distant in the case of the lumbar.

Table S2. Comparative Evaluation Using XGBOOST: Accelerometer vs. Gyroscope Data

	**Accelerometer**		**Gyroscope**	
Sensors	MAE ± SD	r	MAE ± SD	r
Ankle	0.26 ± 0.15	0.94	0.24 ± 0.17	0.93
Lumbar	0.23 ± 0.15	0.96	0.26 ± 0.16	0.90
Sternum	0.30 ± 0.15	0.88	0.30 ± 0.17	0.87
Wrist	0.32 ± 0.20	0.90	0.36 ± 0.21	0.80
Arm	0.30 ± 0.24	0.88	0.39 ± 0.26	0.85

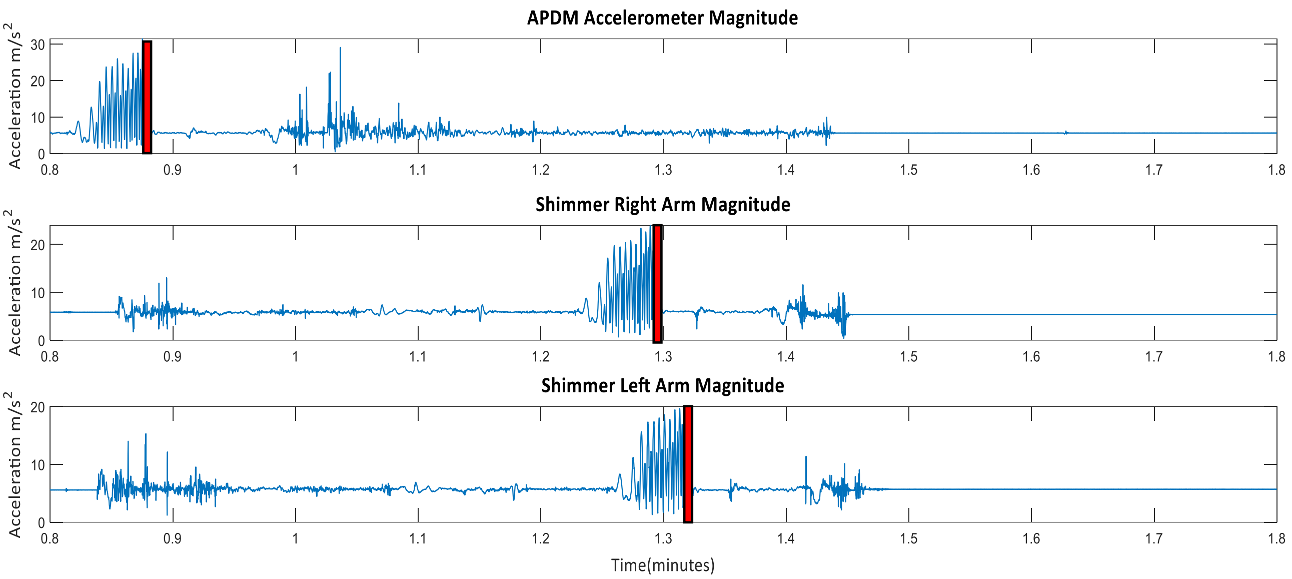

Figure 2A. Before synchronization

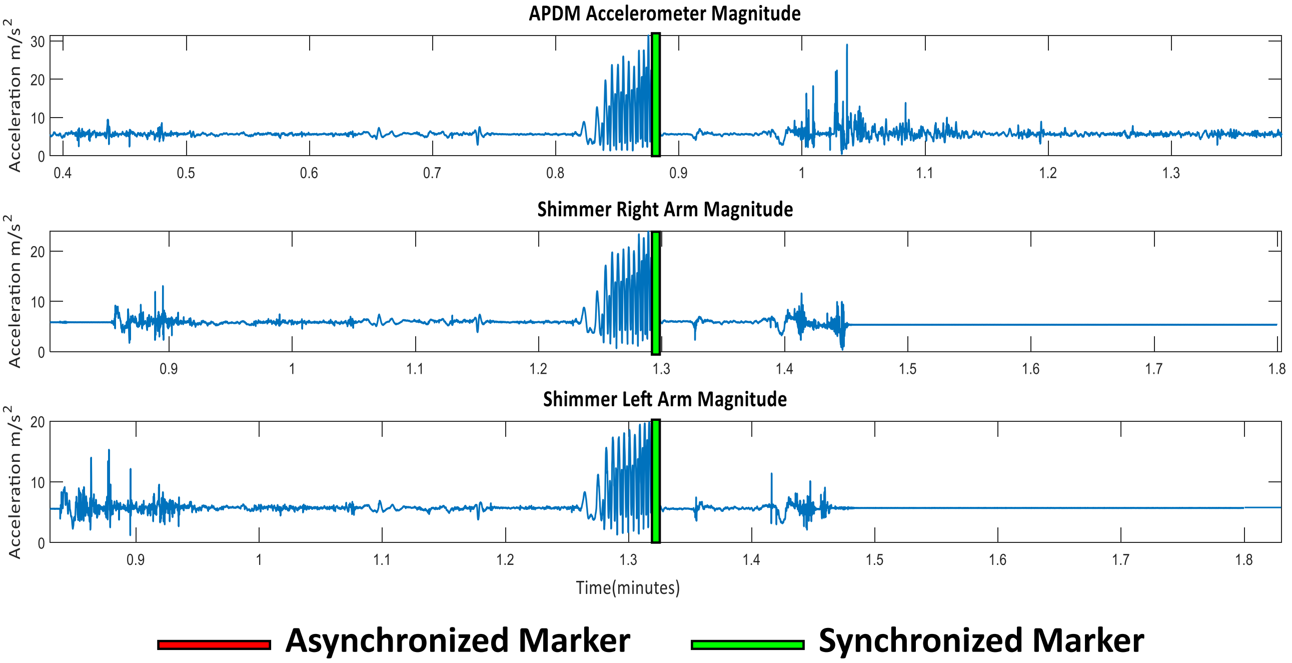

Figure 2B. After synchronization

Figure S2. Synchronization of APDM and Shimmer sensors. **(A)** Depicts the raw signals collected from one APDM sensor and two Shimmer sensors, which were initially shaken to create a recognizable pattern for the synchronization process. **(B)** Illustrates the pre-processing stage, where all sensor signals were synchronized based on this recognizable pattern.

REFERENCES

Cohen H, Blatchly CA, Gombash LL. A study of the clinical test of sensory interaction and balance. *Physical therapy* **73** (1993) 346–351.

Whitney SL, Wrisley DM. The influence of footwear on timed balance scores of the modified clinical test of sensory interaction and balance. *Archives of physical medicine and rehabilitation* **85** (2004) 439–443.

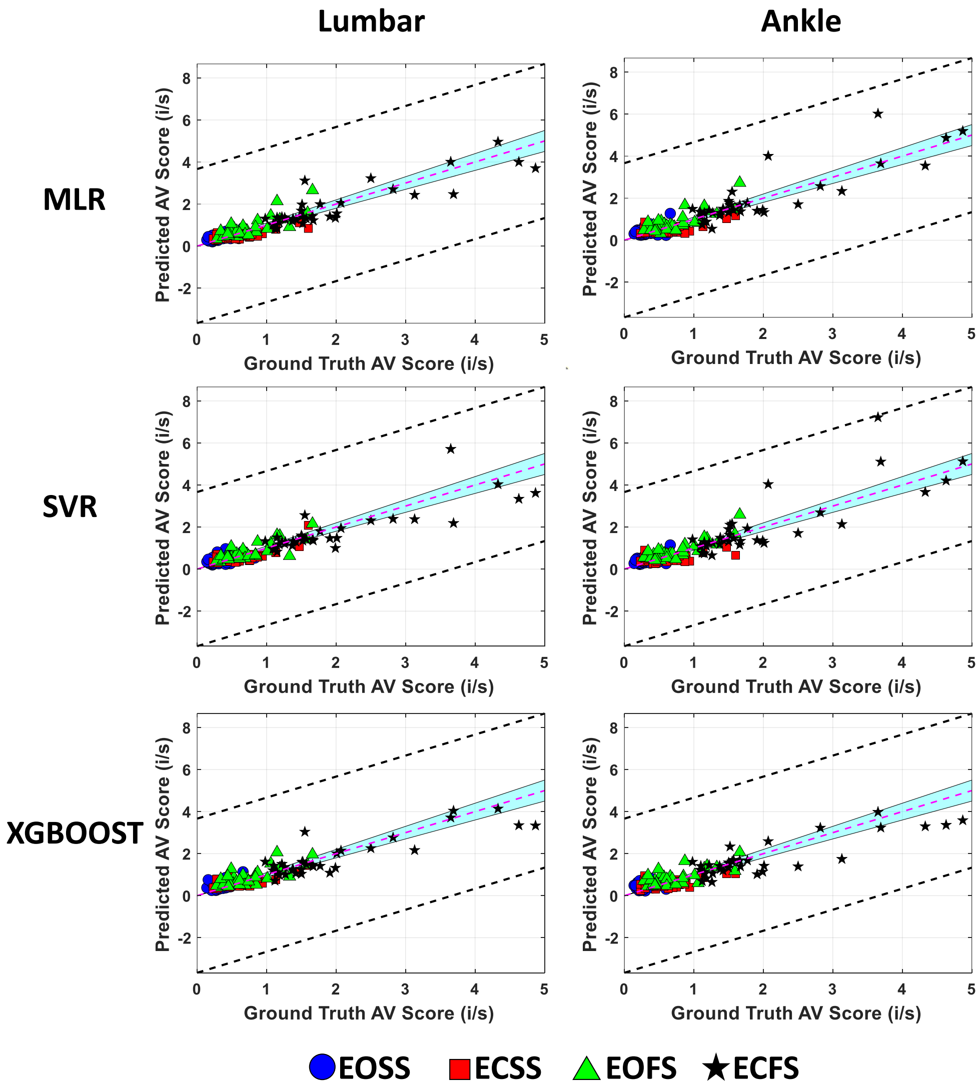

Figure S3. Correlation results between ground truth and predicted m-CTSIB scores using MLR, SVR, and XGBOOST models. The purple and black dashed lines represent a correlation coefficient of one and 95% prediction band, respectively. Scores are reported for four conditions: eyes open-stable surface (EOSS), eyes closed-stable surface (ECSS), eyes open-foam surface (EOFS), and eyes closed-foam surface (ECFS). The abbreviation i/s means inches/second.

Wrisley DM, Whitney SL. The effect of foot position on the modified clinical test of sensory interaction and balance. *Archives of physical medicine and rehabilitation* **85** (2004) 335–338.

Boulgarides LK, McGinty SM, Willett JA, Barnes CW. Use of clinical and impairment-based tests to predict falls by community-dwelling older adults. *Physical therapy* **83** (2003) 328–339.

Horak FB. Clinical measurement of postural control in adults. *Physical therapy* **67** (1987) 1881–1885.

Park MK, Kim KM, Jung J, Lee N, Hwang SJ, Chae SW. Evaluation of uncompensated unilateral vestibulopathy using the modified clinical test for sensory interaction and balance. *Otology & Neurotology* **34** (2013) 292–296.

Freeman L, Gera G, Horak FB, Blackinton MT, Besch M, King L. Instrumented test of sensory integration for balance: a validation study. *Journal of geriatric physical therapy* **41** (2018) 77–84.
